# One Health Index applied to countries in South America

**DOI:** 10.3389/fpubh.2024.1394118

**Published:** 2024-10-08

**Authors:** Alessandra Cristiane Sibim, Wagner Antonio Chiba de Castro, Louise Bach Kmetiuk, Alexander Welker Biondo

**Affiliations:** ^1^Latin-American Institute of Technology, Infrastructure and Territory, Federal University for Latin American Integration (UNILA), Foz do Iguaçu, Brazil; ^2^Graduate College of Veterinary Sciences, Department of Veterinary Medicine, Federal University of Paraná State, Curitiba, Brazil; ^3^Latin-American Institute of Life and Nature Sciences, Federal University for Latin American Integration (UNILA), Foz do Iguaçu, Brazil; ^4^Zoonoses Surveillance Unit, Municipal Secretary of Health, Curitiba, Brazil

**Keywords:** health indicators, socioeconomical factors, ecosystemic services, livestock, political stability

## Abstract

**Introduction:**

The One Health concept has proposed an integrated and unified approach aiming for health balance and enhancement by recognizing the interdependence of human, animal, and environmental health. The COVID-19 pandemic has pushed global One Health initiatives and policy improvement toward preventive measures for future pandemics, particularly of zoonotic origin. Such a scenario may be particularly relevant for South America, which is considered highly vulnerable due to its natural biodiversity superposed to socioeconomic and environmental issues, demanding effective methods and indicators for proper One Health strategies and goals that are aligned with macroregional contexts.

**Methods:**

Accordingly, the present study aimed to assess the One Health Index (OHI) in South American countries, along with potential interactions with socioeconomic indicators. The results obtained using clustering analysis and permutational multivariate analysis of variance (PERMANOVA) have revealed a positive association between the OHI and the Human Development Index (HDI) but not with gross domestic product (GDP).

**Results:**

Although South American countries with political stability, robust investment in health, and progressive policies have shown a higher OHI, better environmental health is not associated with better human and animal health. In addition, although the Amazon biome— spanning 9 of the 12 South American countries—has positively impacted environmental health, this benefit contrasts with the rudimentary local human health systems, highlighting the complexity of One Health within the South American context. The lack of stronger indicators for animal health was also considered an important weak point for a true OHI assessment. Nonetheless, countries with more developed livestock have presented better animal health, which may not reflect an overall animal health indicator, as companion and wildlife animal health indicators were not available.

**Discussion:**

Although lower (within-country) scale analysis such as states and metropolitan areas may better shape internal differences, the study herein has clearly shown One Health inequalities and challenges among South American countries. Equally important, forests and other natural areas in developing countries, particularly the Amazon, should receive incentives to promote sustainable economic growth. This approach would help prevent sacrificing environmental health for the benefit of human and livestock animal health.

## 1 Introduction

One Health has been defined as an integrated and unified approach aiming for a sustainable assessment of human, animal, and environmental health, with a holistic strategy existing long before the term was coined (1). A recent One Health consensus report has considered humans, domestic and wildlife animals, plants, and their ecosystemic environment as intimately connected and interdependent (2), with multidisciplinary and professional integration aiming to better recognize zoonotic emergences from a One Health perspective (3).

The recent COVID-19 pandemic has highlighted the need for a quadripartite agreement to support such initiatives (4). This agreement emphasizes preventing future epidemics through a global understanding of new disease emergence (5) and has been officially approved by the Pan-American Health Organization (PAHO) as a tool for dealing with health threats at the human–animal interface (6). The first action point of this policy resolution aimed to perform an analysis and map the complex interactions among actors and processes in the fields of human, animal, plant, and environmental health in such specific national contexts.

South America has been considered the Americas meridional portion, including 12 sovereign states and two dependent territories (7). Although comprising some of the most important natural reserves worldwide, such as the Amazon Rainforest and Andes, several cultural heritage sites, and large cultivable areas, the South American region remains mostly underdeveloped and highly vulnerable to deforestation, poaching, and zoonotic diseases (8). Although nine South American countries share the Amazon forest, the world’s largest tropical rainforest (9), which provides a significant carbon sink service and helps regulate global climate (10), several socioeconomic and environmental threats (11, 12) have demanded a trans-and multidisciplinary One Health approach to fight such complex challenges (13).

Although widely recognized, the One Health approach has required better and adapted strategies for specific socio-ecological contexts (14, 15). This includes the integration of information technology and statistical analysis to assess environmental and sustainable effectiveness (16), improved climate and environmental comprehension (17), evaluation of risk from current and future natural disasters (18), identification of vulnerable populations and areas (19), and the practical use of the One Health Index itself (20). Accordingly, the present study aimed to assess available indicators of human, animal, and environmental health from a statistical and comparative One Health Index (OHI) perspective of all 12 South American countries.

## 2 Materials and methods

### 2.1 Countries and socioeconomic factors

The present study included all 12 South American countries, namely Argentina, Bolivia, Brazil, Chile, Colombia, Ecuador, Guiana, Paraguay, Peru, Suriname, Uruguay, and Venezuela. The socioeconomic factors of these countries were obtained from official reports and sites and included the Human Development Index (HDI) (21) and the gross domestic product (GDP) *per capita*, based on the purchasing power parity (PPP) (22). The Human Development Index (HDI) has been described as a measure of a country’s well-being and development, combining life expectancy, education, and standard of living, as the geometric mean of normalized indexes for each of these three dimensions (23). The gross domestic product (GDP) *per capita*, based on purchasing power parity (PPP), represents the average value of all goods and services produced by a country in a given year, adjusted for price differences, and expressed in international dollars, providing a more accurate measure of a country’s comparative living standard (24).

### 2.2 Human, animal, and environmental health indicators

Updated indicators for human, animal, and environmental health of all 12 South American countries were selected from the available literature and official reports and sites to construct the One Health Index (Table 1; Supplementary Tables 1–4), based on the One Health Index previously established by our research group (20).

**Table 1:** Indicators for human, animal, and environmental health to construct the One Health Index of all 12 South American countries.

Health category	Performance indicators (PI)	Source	Reference
Human	1. GHS Index	Global Health Security Index	(44)
	2. Social Vulnerability	Multidimensional Vulnerability Index	(29)
	3. Vulnerability	World Risk Report	(45)
Animal	4. Zoonoses	Global Health Security Index	(44)
	5. Pesticides	Food and Agriculture Statistics	(46)
	6. WAHIS*	World Organisation for Animal Health	(40)
Environmental	7. Environmental vulnerability	Multidimensional Vulnerability Index	(29)
	8. Vulnerability to climate changes	Universal Vulnerability Index	(47)
	9. Environmental performance	Environmental Performance Index	(16)

^*^Qualitative information parameters (absence and presence) of each country were divided into: 1. Disease; 2. serotype, subtype, and genotype; 3. animal category; 4. outbreak identifier; and 5. vaccinated.

### 2.3 Data analysis and One Health Index construction

The assessment of each country was based on nine indicators, which were equally distributed into three indicators per health category (Table 1). A performance (ranking) score was attributed to each country for each indicator, with the lowest graded as 1, the highest graded as 12, and the remaining ones graded accordingly (Table 2). The final grade for each country in each health category was the average of the three indicators. Thus, each sampling unit was the composite result of indicators, representing the three weighted grades, corresponding to each of the three health categories, as established and adapted (20). The One Health Index (OHI) of each country was calculated as the average of grades from the three correspondent health categories. It is important to mention that such applied methodology has resulted in composite indexes reflecting a relative panorama of One Health among South American countries, with a comparative rather than absolute OHI.

**Table 2:** Performance (ranking) grades attributed to each South American country, based on the nine performance indicators (PI), which comprise the environmental, animal, and human health categories, along with the grades for each category, expressed as a total.

Countries	Environmental health				Animal health				Human health			
	PI.1	PI.2	PI.3	Total	PI.4	PI.5	PI.6	Total	PI.7	PI.8	PI.9	Total
Argentina	3	1	6	3.3	9	10	8	9.0	10	11	10	10.3
Bolivia	4	4	4	4.0	5	4	10	6.3	2	7	7	5.3
Brazil	6	8	8	7.3	11	12	4	9.0	8	5	4	5.7
Chile	2	2	12	5.3	4	9	7	6.7	12	12	11	11.7
Colombia	11	10	7	9.3	10	11	1	7.3	9	4	1	4.7
Ecuador	12	11	11	11.3	8	8	2	6.0	7	1	3	3.7
Guiana	1	7	2	3.3	1	2	3	2.0	3	6	8	5.7
Paraguay	5	5	5	5.0	6	6	9	7.0	5	2	9	5.3
Peru	9	3	3	5.0	7	7	11	8.3	11	9	2	7.3
Suriname	10	9	9	9.3	3	1	5	3.0	4	8	6	6.0
Uruguay	8	12	1	7.0	12	5	6	7.7	6	10	12	9.3
Venezuela	7	6	10	7.7	2	3	12	5.7	1	3	5	3.0

### 2.4 Ranking (score) according to socioeconomic factors

Countries were ordered according to their grades of each health category and analyzed using principal component analysis (PCA), which classified countries using cluster analysis, exploring 1. One Health Index, 2. Human Development Index (HDI), and 3 gross domestic product (GDP) *per capita*, based on purchasing power parity. Statistical significance for country clusters in each factor was assessed using clustering analysis and permutational multivariate analysis of variance (PERMANOVA) (25), based on the three first coefficients of principal components, obtained from each PCA. A *p*-value less than 0.05 was considered significant. All statistical analyses were performed in the statistical environment R (26).

## 3 Results

**Figure 1:**
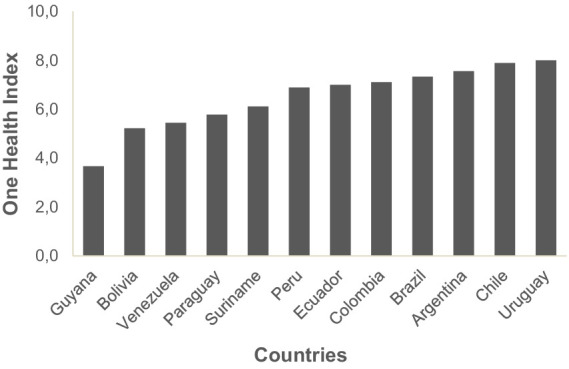
Graphic of One Health Index grading of South American countries.

**Figure 2:**
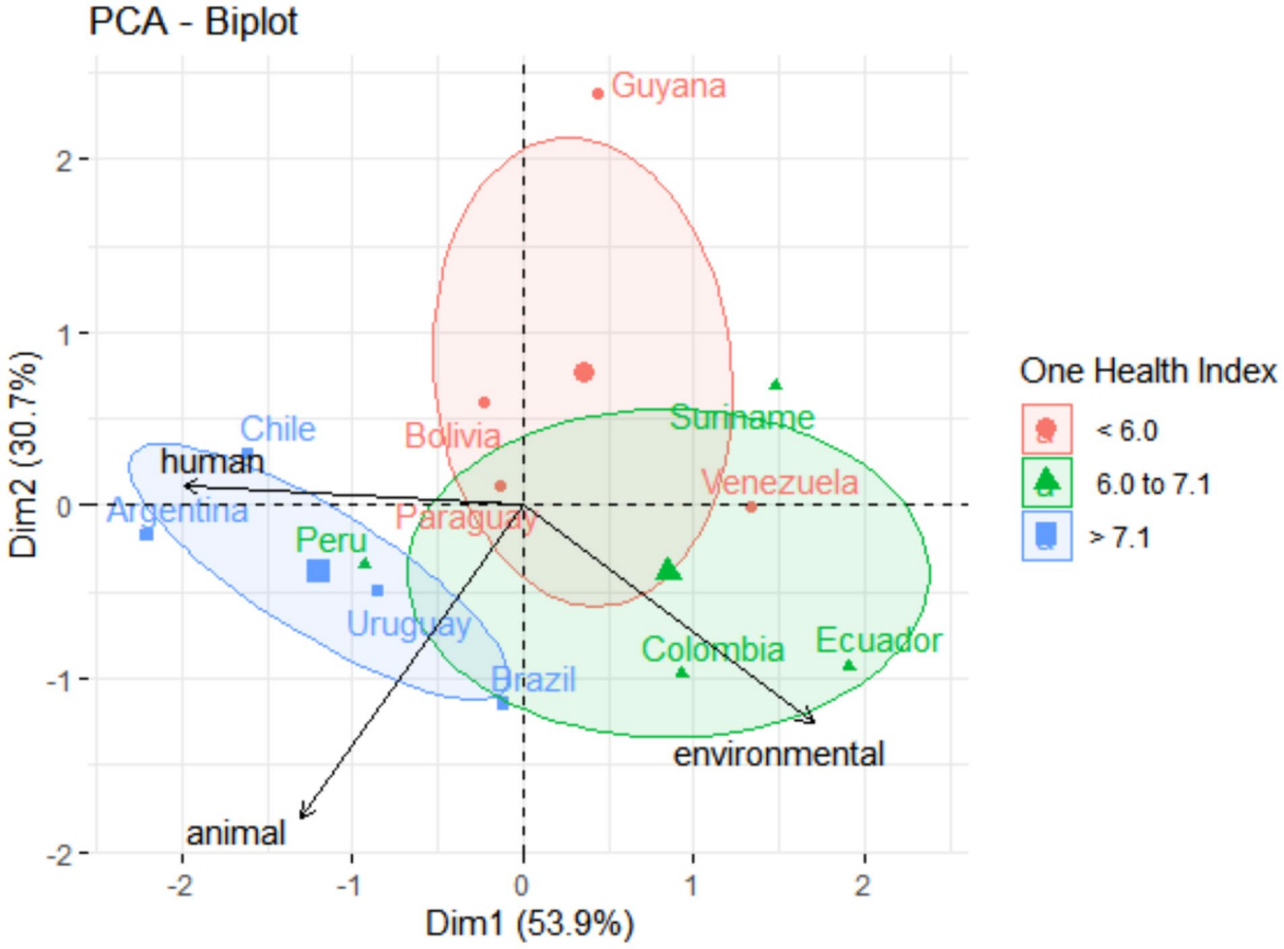
Graphic of principal components analysis (PCA) showing the influence of (human, animal, and environmental) health variables on all the South American countries. Colors and ellipses circling the country groups represent the confidence ellipsis, which delimited country clusters according to grading intervals of One Health.

The results of One Health for each South American country were obtained, gathered, and presented (Figure 1; Supplementary Table 5). Overall, Uruguay (8.0), Chile (7.9), and Argentina (7.6) presented the highest grade of One Health. Guiana (3.7), Bolivia (5.2), and Venezuela (5.4) presented the lowest grade of One Health. The principal component analysis, presented in Figure 2, illustrates the significant differences among South American countries based on human, animal, and environmental health categories, as determined by the One Health grading (PERMANOVA; *F* = 3.6305; *p* = 0.009). Countries with higher One Health grades (Uruguay, Chile, and Argentina) were grouped to the left of the graphic.

**Figure 3:**
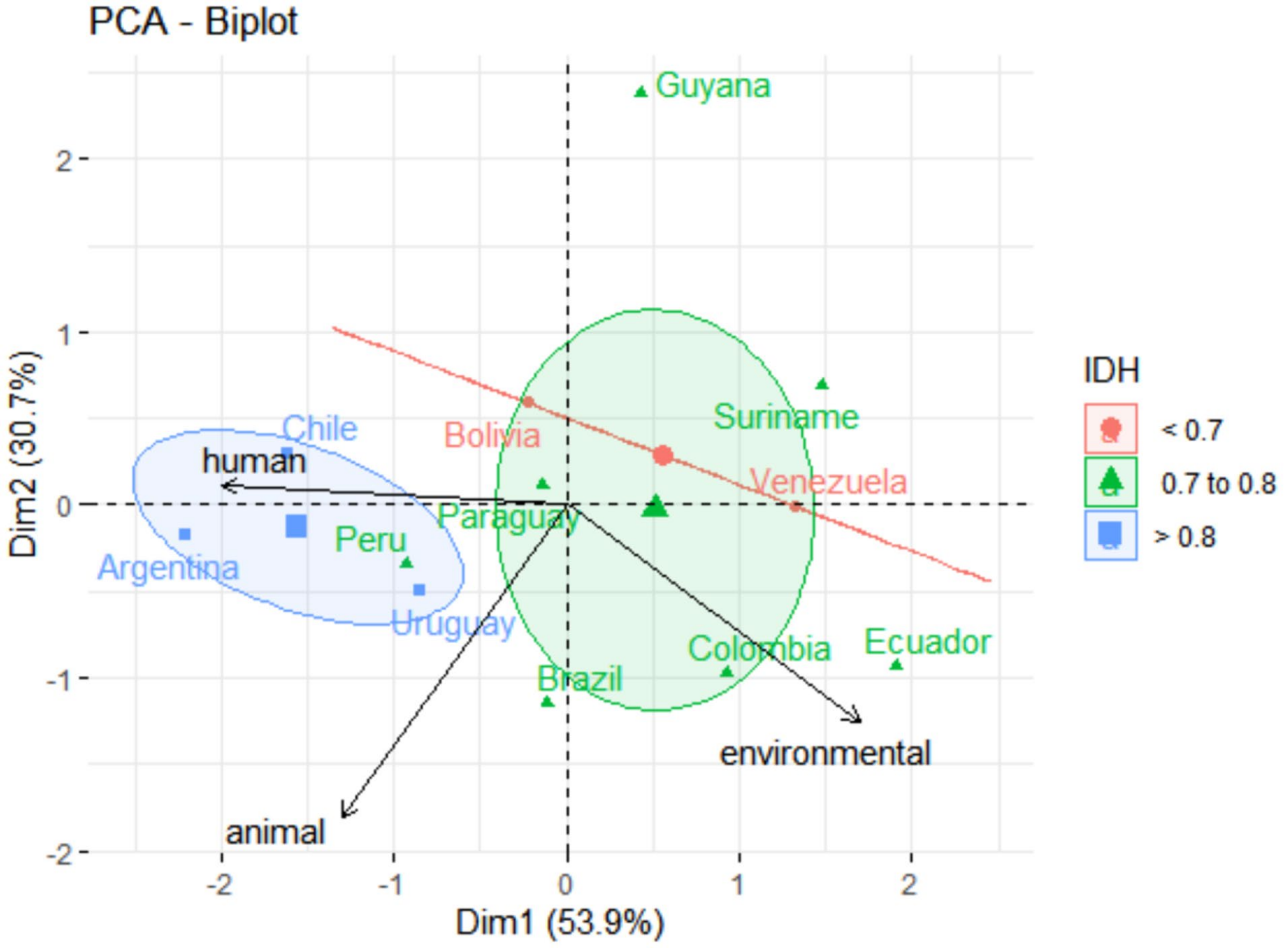
Graphic of principal component analysis (PCA) showing the influence of (human, animal, and environmental) health variables on all the South American countries. Colors and ellipses circling the country groups represent the confidence ellipsis, which delimited country clusters according to the higher Human Development Indexes (HDIs).

**Figure 4:**
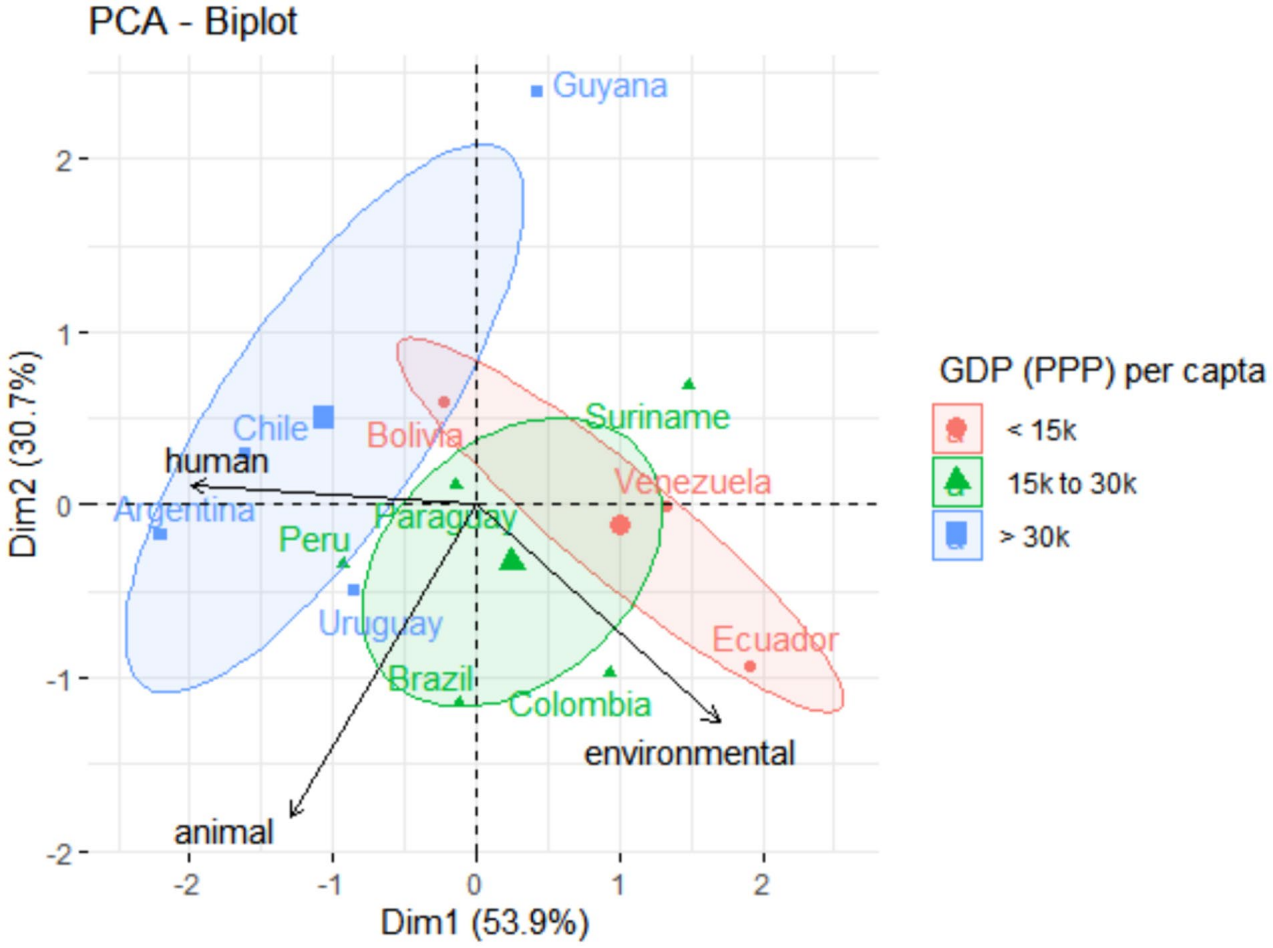
Graphic of principal component analysis (PCA) showing the influence of (human, animal, and environmental) health variables on the South American countries. Colors and ellipses circling the country groups represent the confidence ellipses, which delimited country clusters according to the gross domestic product (GDP) *per capita*, based on purchasing power parity (in U$).

The same pattern can be observed in significant differences among countries on health categories compared to the Human Development Index (HDI) (Figure 3). Chile (0.855), Argentina (0.842), and Uruguay (0.809) (countries grouped to the left of the graphic) also presented higher HDI (PERMANOVA; *F* = 4.9113; *p* = 0.001). However, no significant difference was observed for South American countries and gross domestic product (GDP) *per capita* based on purchasing power parity (PPP) (PERMANOVA; *F* = 2.0781; *p* = 0.10) (Figure 4). Guyana presented, at the same time, the highest GDP-PPP (U$ 60,650) and the lowest OHI (3.7).

## 4 Discussion

The results indicated that South American countries with higher Human Development Indexes (HDIs) also had higher One Health Indexes (OHIs), which are composed of various education and health indicators. However, the results also showed no significant association between the One Health Index and gross domestic product (GDP) *per capita*. This suggests that the holistic nature of One Health is better explained by the HDI’s social approach rather than by an economic index such as GDP, even when adjusted for purchasing power. Thus, despite the importance of explaining health in several contexts (27, 28), the economic factor was not the sole determinant of One Health.

The study herein showed that South American countries with higher environmental health also presented lower human and animal health. While Ecuador (1st), Colombia (2nd), Suriname (3rd), and Venezuela (4th) presented the highest scores in environmental health, their performance in animal health (Ecuador 9th and Suriname 11th) and human health (Colombia 10th, Ecuador 11th, and Venezuela 12th) was relatively lower. The environmental health indicators explored aspects such as climate change mitigation, air quality, biodiversity and ecosystemic services, fishing, and water resources (16), as well as vulnerability to natural disasters (29) and risk areas (30). These South American countries have their territory overlapped by the Amazon forest (42% of Colombia, 48% of Ecuador, and 94% of Suriname), with most populations living in close contact with natural areas (31). Thus, the ecosystemic services provided by the rainforest may have a favorable impact on applied performance indicators of environmental health. The Amazon forest has been threatened by illegal human activities such as logging, mining, and fires. Notably, the southern and southeastern Brazilian regions of the Amazon have experienced increasing soil erosion and a 7% deforestation rate (411,857 km^2^) between 1960 and 2019, due to expanding agriculture and livestock activities (32). The annual Brazilian Amazon deforestation has surpassed 13,000 km^2^ from 2019 to 2021, which represents an increase of 56.6% when compared to 2016–2018 (33). Such an increase was reportedly associated with a government attempt to promote environmental sustainability through the agribusiness-based economy in the southern and southeastern Brazilian Amazon, which culminated in land grabbing, conflicts, and deforestation (33). In addition, modeling studies have indicated an increase of 4°C in temperature or deforestation exceeding 40% as two “tipping points” of irreversible changes for biodiversity and ecosystems of the Amazon forest (34). In such a scenario, recent studies have advocated for sustainable development in the Amazon, based on the non-use of natural resources, accompanied by an effort to improve ecosystem resilience (34, 35). Thus, considering the environmental health role for a better One Health, forests and other natural areas of developing countries (particularly the Amazon forest) should receive incentives for sustainable economic growth, preventing the sacrifice of environmental health for the benefit of human and livestock animal health. Although the data presented reflect the most recently available information, providing only a current temporal snapshot, the historical overall development situation in South America and its negative impact on the Amazon Rainforest as a side effect consequence over time should be considered a warning for a truly sustainable and healthy development of the region.

Despite having large natural areas, South American countries have deep health system limitations, with recent trajectories of health privatization and increased access inequalities to health services (36), political crises affecting the quality of services provided (37), and shortage of human resources (38). Thus, South American countries with political stability, higher investment in human health, and progressive political characteristics have been placed at a higher level of the One Health Index (OHI). The four countries with the highest scores in the OHI (Uruguay, Chile, Argentina, and Brazil) were among the five countries with the highest investments in human health *per capita* (39), presenting solid democratic political regimes throughout the last decades.

A limitation of this study is the difficulty in accessing animal health indicators, which were used along with several standard composite indexes of human and environmental health. Only one integrated animal health index was found at the global level, the Animal Protection Index (API), recently provided by the World Animal Protection (WAP), a non-profit organization. However, such an index was not used herein, as only 7 of 12 (58.3%) South American countries presented available API grades. Thus, only indirect performance indicators of animal health were explored, such as zoonoses, pesticides (harmful to natural biota), and a selection of livestock indicators obtained from the World Animal Health Information Systems (WAHIS), a database maintained by the World Organisation of Animal Health (40). Thus, the animal health approach was based exclusively on livestock health (and not welfare), excluding analysis of both companion and wildlife animal health.

A previous One Health Index (OHI) study conducted at the city level Curitiba, the eighth biggest metropolitan area of Brazil, has also shown difficulties in obtaining animal health indicators (20, 41). In this study, qualitative (yes or no) indicators were used, assessing only companion animal health, including education and neutering/spaying programs, animal hoarder monitoring, enforcement against animal cruelty, microchipping, and adoption of abandoned pets. Such a lack of comprehensive and reliable data in the present study may have biased animal health as livestock health only. Thus, indicators may have rewarded agricultural performance, such as control of animal diseases and conscient use of pesticides. In such a scenario, South American countries with advanced livestock production, such as Brazil, Argentina, and Uruguay, were among the highest scorers for animal health in South America. Thus, further efforts and studies should focus on providing reliable animal health indexes for livestock, companion animals, and wildlife, which could then be used for comparisons at city, state, country, and continental levels.

As another limitation, the present study has assessed information at international official organizations, at country level, available in official languages of South American countries including English (Guiana), French (French Guiana), Portuguese (Brazil), and Spanish (all others), with exception of Dutch in Suriname and official native indigenous languages such as Guarani in Bolivia and Paraguay, Aymara in Bolivia and Peru, and Quechua in Bolivia, Ecuador and Peru. In addition, information obtained, particularly in large territorial countries, may not represent the within-country inequalities among states and provinces, or even among cities within the same state or province. In addition, as well-known, the largest South American metropolitan areas have been characterized by deep within-city inequalities, such as São Paulo, Buenos Aires, and Santiago cities (42). As the One Health Index applied at the country level may be impaired by inequalities and disparities at the state and city levels, further studies should compare and contrast One Health Index patterns across local, metropolitan, and regional regions within countries.

Despite the authors’ recognition of the importance of temporal and spatial analyses, the data herein did not support a temporal analysis because the surveys only included the most recent available data, providing a current temporal snapshot. Although the data herein did not support spatial analysis, the discussion was focused on countries overlying the Amazon rainforest biome, which is important for current analysis and further research. The authors also acknowledge that the data herein may be insufficient to explore smaller spatial scales (such as large metropolitan areas), which would enhance the understanding of the One Health landscape in South America.

Finally, although in the highest available resolution and with bigger letters and captions, the figures presented in the present study have a standard outcome layout provided by the Tidyverse, Stats, and Factoextra statistical packages, which were used to build them.

Other One Health assessments have been recently reported, focusing on interdisciplinary setting effectiveness, with assessment tools of a calculated hexagon presented as OH-index (OHI) and OH-ratio (OHR) in spider diagrams, along with Theory of Change (TOC) as indicator for measurement of expected results, comparing One Health with conventional health initiatives (43). In addition, a Global One Health Index (GOHI) based on a three-layer framework has been proposed for the evaluation of One Health structure, process, and outcome (51). Although presenting important contributions to One Health assessment, both studies may lack the practical approach presented by the One Health Index (OHI) applied herein (20), which has combined several health indexes within human, animal, and environmental components, providing holistic and comparative strengths and weaknesses among municipalities.

The holistic One Health Index (OHI) approach herein has provided a better understanding of health as a whole in South American countries, contextualized by the Human Development Index (HDI) and contrasted by the gross domestic product (GDP) *per capita*. The higher grades of environmental health in some South American countries have not necessarily indicated better human and animal health. Progressive policies, consistent investments in human health, and political stability were important factors associated with higher One Health grades. The limitations and lack of reliable indicators, particularly for environmental and animal health, have highlighted the need for better indexes worldwide. Although within-country inequalities may have influenced the results of the present study, this is the first attempt to compare One Health in such a practical manner, and further studies should address local, metropolitan, and regional regions within countries in South America and other continents.

## Data Availability

The original contributions presented in the study are included in the article/Supplementary material, further inquiries can be directed to the corresponding authors.

## Supplementary material

SUPPLYMENTARY TABLE 1 Socioeconomic data of the countries, represented by the Human Development Index (HDI) (23) and the Gross Domestic Product per capita adjusted by purchasing power parity (in dollars) (48) of South American countries, as well as the considered classes.

SUPPLYMENTARY TABLE 2 Raw values of the environmental health performance parameters of South American countries. EV_MVI = Environmental Vulnerability, Multidimensional Vulnerability Index (UN, 2023); PVCCI_UVI = Physical Vulnerability to Climate Change Index, Universal Vulnerability Index (49); EPI = Environmental Performance Index (50).

SUPPLYMENTARY TABLE 3 Raw values of the animal health performance parameters of South American countries. ZD_GHS = Zoonosis, Global Health Security Index (Bell and Nuzzo, 2021); PEST_FAO = Pesticides (kg*ha-1), Food and Agriculture Statistics; WAHIS = Parameters, World Organisation for Animal Health.

SUPPLYMENTARY TABLE 4 Raw values of the human health performance parameters of South American countries. SV_MVI = Social Vulnerability, Multidimensional Vulnerability Index; VUL_WRR = Vulnerability, World Risk Report (51); GHS = Global Health Security Index.
