# Supplementary material for: One Health Index applied to countries in South America
Source: Front Public Health. 2024 Oct 8;12:1394118. doi: 10.3389/fpubh.2024.1394118 (PMC11495393; doi:10.3389/fpubh.2024.1394118)
Supplement: Supplementary file 1 [file Table_1.docx]

Supplementary Material

# Supplementary Table 1. Socioeconomic data of the countries, represented by the Human Development Index (HDI) (1) and the Gross Domestic Product per capita adjusted by purchasing power parity (in dollars) (2) of South American countries, as well as the considered classes.

| **Countries** | **HDI** | **Classes** | **GDP (PPP)** | **Classes** |
| --- | --- | --- | --- | --- |
| Argentina | 0.842 | High | 31314 | High |
| Bolivia | 0.692 | Low | 14906 | Low |
| Brazil | 0.754 | Average | 19948 | Average |
| Chile | 0.855 | High | 31496 | High |
| Colombia | 0.752 | Average | 21056 | Average |
| Ecuador | 0.74 | Average | 15931 | Low |
| Guyana | 0.714 | Average | 60650 | High |
| Paraguay | 0.717 | Average | 20451 | Average |
| Peru | 0.762 | Average | 19587 | Average |
| Suriname | 0.73 | Average | 21016 | Average |
| Uruguay | 0.809 | High | 32744 | High |
| Venezuela | 0.691 | Low | 8030 | Low |

Supplementary Table 2. Raw values of the environmental health performance parameters of South American countries. **EV_MVI** = Environmental Vulnerability, Multidimensional Vulnerability Index (UN, 2023); **PVCCI_UVI** = Physical Vulnerability to Climate Change Index, Universal Vulnerability Index (3); **EPI** = Environmental Performance Index (4).

| **Countries** | **EV_MVI** | **PVCCI_UVI** | **EPI** |
| --- | --- | --- | --- |
| Argentina | 43.6 | 43.43 | 41.1 |
| Bolivia | 43.4 | 33.89 | 40.1 |
| Brazil | 34.2 | 31.58 | 43.6 |
| Chile | 53.4 | 40.53 | 46.7 |
| Colombia | 28.9 | 25.28 | 42.4 |
| Ecuador | 28.6 | 24.54 | 46.5 |
| Guyana | 58.9 | 32.38 | 38.5 |
| Paraguay | 39.7 | 33.45 | 40.9 |
| Peru | 31.8 | 37.48 | 39.8 |
| Suriname | 30.5 | 29.24 | 45.9 |
| Uruguay | 31.9 | 21.75 | 37.4 |
| Venezuela | 32.3 | 32.65 | 46.4 |

Supplementary Table 3. Raw values of the animal health performance parameters of South American countries. **ZD_GHS** = Zoonosis, Global Health Security Index; **PEST_FAO** = Pesticides (kg*ha-1), Food and Agriculture Statistics; **WAHIS** = Parameters, World Organisation for Animal Health.

| **Countries** | **ZD_GHS** | **PEST_FAO** | **WAHIS** | | | | |
| --- | --- | --- | --- | --- | --- | --- | --- |
|  |  |  | **a** | **b** | **c** | **d** | **e** |
| Argentina | 46.5 | 488 | 1 | 0 | 1 | 1 | 1 |
| Bolivia | 26.2 | 359 | 1 | 1 | 0 | 0 | 0 |
| Brazil | 58.3 | 958 | 1 | 1 | 1 | 1 | 1 |
| Chile | 18.9 | 719 | 1 | 0 | 1 | 1 | 1 |
| Colombia | 48.8 | 1513 | 1 | 1 | 1 | 1 | 1 |
| Ecuador | 45.7 | 1403 | 1 | 1 | 0 | 0 | 1 |
| Guyana | 1.6 | 1013 | 1 | 0 | 0 | 0 | 1 |
| Paraguay | 31 | 468 | 1 | 1 | 0 | 1 | 0 |
| Peru | 43.6 | 187 | 1 | 1 | 0 | 0 | 1 |
| Suriname | 5.6 | 792 | 0 | 0 | 0 | 0 | 0 |
| Uruguay | 76.9 | 773 | 1 | 0 | 0 | 1 | 1 |
| Venezuela | 2.9 | 119 | 1 | 0 | 1 | 0 | 0 |

WAHIS parameters: a) Disease, b) Serotype/Subtype/Genotype, c) Animal Category, d) Outbreak_id, e) Vaccinated.

Supplementary Table 4. Raw values of the human health performance parameters of South American countries. SV_MVI = Social Vulnerability, Multidimensional Vulnerability Index; VUL_WRR = Vulnerability, World Risk Report (5); GHS = Global Health Security Index.

| **Countries** | **SV_MVI** | **VUL_WRR** | **GHS** |
| --- | --- | --- | --- |
| Argentina | 25.9 | 19.18 | 54.4 |
| Bolivia | 38.9 | 25.30 | 29.9 |
| Brazil | 41.8 | 28.47 | 51.2 |
| Chile | 10.7 | 15.37 | 56.2 |
| Colombia | 51.3 | 44.93 | 53.2 |
| Ecuador | 67.3 | 38.15 | 50.8 |
| Guyana | 40.8 | 25.14 | 30.8 |
| Paraguay | 63.2 | 20.23 | 40.3 |
| Peru | 36.3 | 39.22 | 54.9 |
| Suriname | 37 | 25.33 | 35 |
| Uruguay | 30.6 | 14.96 | 40.3 |
| Venezuela | 61.1 | 28.22 | 20.9 |

Supplementary Table 5. One Health Indices of South American countries.

| **Countries** | **OH index** |
| --- | --- |
| Argentina | 7.6 |
| Bolivia | 5.2 |
| Brazil | 7.3 |
| Chile | 7.9 |
| Colombia | 7.1 |
| Ecuador | 7.0 |
| Guyana | 3.7 |
| Paraguay | 5.8 |
| Peru | 6.9 |
| Suriname | 6.1 |
| Uruguay | 8.0 |
| Venezuela | 5.4 |

Supplementary Table 6. Projections on the Principal Component Analysis axes of South American countries, according to their scores in the three health dimensions (environmental, human, and animal).

| **Countries** | **X-axis** | **Y-axis** | **Z-axis** |
| --- | --- | --- | --- |
| Argentina | -2.20369 | -0.17605 | -0.07484 |
| Bolivia | -0.22698 | 0.590582 | -0.85308 |
| Brazil | -0.11447 | -1.14518 | -0.45529 |
| Chile | -1.61709 | 0.292471 | 1.125731 |
| Colombia | 0.931876 | -0.97359 | -0.00425 |
| Ecuador | 1.909661 | -0.92813 | 0.391575 |
| Guyana | 0.42978 | 2.383216 | -0.19368 |
| Paraguay | -0.14022 | 0.118389 | -0.73905 |
| Peru | -0.92605 | -0.34781 | -0.41207 |
| Suriname | 1.482084 | 0.691562 | 1.078681 |
| Uruguay | -0.85278 | -0.49716 | 0.695065 |
| Venezuela | 1.327879 | -0.00831 | -0.5588 |

References

1. Nations U. Human Development Report 2023-24 [Internet]. Human Development Reports. United Nations; 2024 Mar [cited 2024 Jul 28]. Available from: https://hdr.undp.org/content/human-development-report-2023-24

2. IMF DataMapper [Internet]. [cited 2024 Jul 28]. Available from: https://www.imf.org/external/datamapper/profile

3. The Commonwealth Universal Vulnerability Index.pdf [Internet]. [cited 2024 Jul 28]. Available from: https://sdgs.un.org/sites/default/files/2021-09/The%20Commonwealth%20Universal%20Vulnerability%20Index.pdf

4. Environmental Performance Index [Internet]. [cited 2024 Jul 28]. Environmental Performance Index. Available from: https://epi.yale.edu/

5. WorldRiskReport_2023_english_online.pdf [Internet]. [cited 2024 Jul 28]. Available from: https://weltrisikobericht.de/wp-content/uploads/2024/01/WorldRiskReport_2023_english_online.pdf
